# The changing landscape of interventional cardiology

**DOI:** 10.18632/aging.102005

**Published:** 2019-05-29

**Authors:** Richard J. Jabbour, Azeem Latib

**Affiliations:** 1 Imperial College, London, London, United Kingdom; 2 Interventional Cardiology Unit, EMO-GVM Centro Cuore Columbus, Milan, Italy; 3 Interventional Cardiology Unit, San Raffaele Scientific Institute, Milan, Italy; 4 Division of Cardiology, Department of Medicine, University of Cape Town, Cape Town, South Africa

**Keywords:** transcatheter aortic valve implantation, aortic stenosis

Transcatheter aortic valve replacement (TAVR) has changed the landscape of interventional cardiology and is now the treatment of choice for patients with severe aortic stenosis and elevated surgical risk [1]. TAVR was once a procedure deemed only suitable for non-surgical extreme risk patients, but since the first implantation in 2002, several hundred thousand procedures have been performed worldwide and randomized studies have now reported the superiority of TAVR over conventional open heart surgery, even in low risk surgical patients [1,2]. Whilst early procedures were commonly associated with severe periprocedural complications and protracted in-hospital stays, operator familiarity and equipment design improvements have drastically improved outcomes. For example, lower profile devices and delivery systems, pre-operative computed tomography, and improvements in vascular closure devices have now made TAVR a “minimal” procedure with many patients often discharged after an overnight stay [3]. However, due to the intrinsic nature of TAVR, and the displacement of the old diseased valve by the new percutaneously implanted valve, certain complications including ostial coronary artery obstruction continue to persist and are often deadly with mortality rates of up to 50% [4]. It is generally recognised to occur acutely during the index procedure and predominantly because of obstruction of the coronary ostia by the displaced native valve leaflets and classically in patients with high risk features (previous bioprosthetic valve, narrow sinuses of Valsalva, low coronary heights) [4]. Recently however, data were reported from a large registry (consisting of 18 international centres and data from 17,092 TAVR procedures over 11 years) regarding delayed coronary obstruction (DCO), a new complication in which the coronary obstruction occurs after the index procedure and in some cases months and years after the index procedure [5]. The reported incidence was low (0.22%, n=38) but this may well be under-reported; for example, if sudden cardiac death was the first manifestation of DCO, it may go undiagnosed if no autopsy is performed. Additionally, patients would be protected from DCO if they have had prior coronary artery bypass surgery. The finding and description of DCO is important since originally TAVR was implanted in high or extreme risk patients with relatively low survival rates (up to 30% mortality at 1 year in high risk surgical patients). This means that patients could have died prior to the development of DCO. As understandably cardiologists and patients are keen to continue embracing TAVR over conventional surgery and there is a drive to implant in lower surgical risk patients, it is important to reflect and gather data on this new phenomenon. Since the publication of the original report, there have been concerns raised that DCO may even be the structural equivalent of the feared complication of late stent thrombosis (seen 1-12 months post implantation), that was deadly and discovered only after drug eluting stents had been rapidly adopted throughout the world (1-2% incidence with mortality rate of up to 50%) [6]. However, it must be stated that the incidence of DCO from the multicentre registry was lower at 0.22% and TAVR clearly has prognostic benefit in the treatment of severe AS. In addition, 2 out of 3 DCO patients had one classical risk factor for acute coronary obstruction (valve-in-valve procedure, narrow sinus of Valsalva, low coronary heights) and therefore was more likely to occur in patients with high-risk anatomies for obstruction. However, since a significant proportion of patients (one third) were not high risk for coronary obstruction, future studies are needed to define new risk factors which could include calcium distribution, leaflet length and morphology. In addition, future generation valves could be designed to minimise obstruction from occurring. The multiple ongoing research studies should specifically define DCO as a recognised complication so that further information can be obtained to monitor the incidence and understand the condition in greater detail.
